# Editorial: Hypertension and cardiorenal syndrome and their relationship with aging: friends and foes

**DOI:** 10.3389/fragi.2025.1760202

**Published:** 2025-12-15

**Authors:** Humberto Muzi-Filho, Adalberto Vieyra

**Affiliations:** 1 Center for Research in Precision Medicine, Carlos Chagas Filho Institute of Biophysics, Federal University of Rio de Janeiro, Rio de Janeiro, Brazil; 2 National Center for Structural Biology and Bioimaging/CENABIO, Federal University of Rio de Janeiro, Rio de Janeiro, Brazil; 3 Graduate Program in Translational Biomedicine/BIOTRANS, Grande Rio University/UNIGRANRIO, Duque de Caxias, Brazil

**Keywords:** aging, cardiorenal syndrome, hypertension, mental health, metabolic biomarkers, non-invasive markers, self-care practices

Hypertension and cardiorenal syndrome are two risk factors in elderly populations (Jarocki et al., 2025), reflecting the dual nature of friends and foes. As a friend, in the early stages of life, adaptive mechanisms in the cardiovascular system compensate for initial hemodynamic stress. However, prolonged stimulation of the Renin-Angiotensin-Aldosterone System and the autonomic nervous system can transform this “friend” into a severe “foe”, provoking deleterious effects on multiple organs, such as the heart and the kidneys, establishing the basis for hypertension and cardiorenal syndrome (Ziegler et al., 2025). In this Research Topic, recently published studies have broadened the understanding of how physiological, psychological, and behavioral factors converge to interact with hypertension and cardiorenal syndrome in hypertensive patients, highlighting the interaction between aging, vascular remodeling, and changes at the renal level, and demonstrating that their consequences go far beyond the numbers expressed on the sphygmomanometer (Kebede et al., 2024; Shangguan et al., 2024; Dai et al., 2025; Du et al., 2025; Fang et al., 2025; Wang et al., 2025; Zeng et al., 2025) (Figure 1).

**FIGURE 1:**
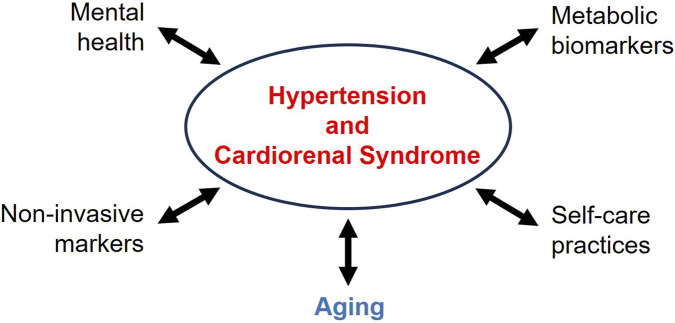
Beyond the numbers expressed on the sphygmomanometer.

Cardiorenal syndrome involves a bidirectional interaction between the heart and kidneys, in which dysfunction in one organ accelerates impairment in the other (Ronco, 2011). Aging can amplify the pathogenesis of cardiorenal syndrome by promoting vascular stiffening, endothelial dysfunction, and impaired maintenance of body homeostasis. In this context, the usefulness of new non-invasive markers, such as estimated pulse wave velocity (ePWV) and shear wave elastography (SWE), is increasingly recognized for early detection of subclinical renal lesions (Dai et al., 2025; Zeng et al., 2025). In the published manuscript on this Research Topic, the increase in ePWV correlates with albuminuria in hypertensive patients, indicating that arterial stiffness contributes to glomerular microvascular stress and early renal impairment, even before changes in renal biomarkers such as serum creatinine (Zeng et al., 2025). Similarly, the SWE study revealed that renal cortical stiffness increases with the progression of hypertension, correlating with the progressive and silent nature of hypertensive nephropathy (Dai et al., 2025). These two studies highlight the importance of studying vascular and renal imaging methods in routine geriatric cardiovascular assessment.

Another element of interest in monitoring hypertensive patients is the assessment of metabolic markers. The manuscript by Du et al. (2025) showed, in a 7-year longitudinal study, that sarcopenia, by itself, is not significantly associated with the development of hypertension in middle-aged and elderly adults. However, obesity associated with sarcopenia significantly increases the risk of developing hypertension, deserving attention in public health strategies aimed at the elderly population. Furthermore, Du et al. (2025) also discuss that obesity leads to increased chronic inflammation, activating the Renin-Angiotensin-Aldosterone System and oxidative stress pathways, culminating in the onset of hypertension, and that sarcopenia not associated with obesity would not trigger these mechanisms. In another study, the Hemoglobin Glycation Index (HGI) is proposed as a biomarker of cardiovascular mortality in hypertensive patients, reflecting data beyond average blood glucose levels (Shangguan et al., 2024). This study also suggests that states of hypoglycation and hyperglycation can confer an elevated cardiovascular risk, correlating with metabolic impairment in older adults. Additionally, lifestyle elements such as diet, physical activity, and adherence to pharmacological treatment are central to the results obtained (Shangguan et al., 2024). In another study, more than 50% of Ethiopian patients reported incorrectly practicing self-care, being influenced by educational issues, little knowledge about hypertension and stress handling, and social inequalities (Kebede et al., 2024). Thus, the study suggested that psychosocial behavioral factors act in conjunction with physiological stressors in the course of arterial hypertension, with particular attention to elderly patients.

Another important issue in the management of hypertension is mental health. A meta-analysis demonstrated that depression in elderly hypertensive patients is associated with a 30%–40% increase in mortality, with increased prevalence mainly among women (Fang et al., 2025). The interaction between mental health and cardiovascular compromise can be assessed through neurohumoral activation, systemic inflammation, endothelial dysfunction, and low adherence to pharmacological treatment. Furthermore, the study by Wang et al. (2025) showed that life satisfaction correlates with subjective perception of health, economic situation, sleep quality, diet, and social interactions in elderly patients, indicating that social and subjective perceptions may have a critical influence on the clinical management of hypertension.

The clinical message is clear: treating hypertension and cardiorenal syndrome alone is insufficient. In this context, the articles published in this Research Topic contribute to understanding the pathophysiology of hypertension and cardiorenal syndrome in aging, encompassing underlying aspects such as metabolic changes, mental health, and behaviors related to personal health (Figure 1). Given that the kidney-heart axis is sensitive to arterial stiffening and age-related metabolic changes, the early identification of susceptible patients through imaging (SWE, ePWV) and biochemical markers (HGI, albuminuria) is fundamental, potentially mitigating progression to chronic renal and cardiac diseases. In this context, the evaluation of the development of obesity and metabolic syndrome, associated or not with sarcopenia, is also an important field in understanding the progression of hypertension. Furthermore, behavioral and psychological interventions, including the diagnosis of depression and the promotion of personal life satisfaction, can be helpful in the therapy of hypertension. Thus, the understanding of these interactions is essential as population aging and the prevalence of chronic multimorbidities increase (Figure 1). In conclusion, integrating these assessments into population screening can contribute to early detection in adults and the elderly, support public health policies to prevent and treat hypertension and cardiorenal syndrome, and improve the quality of life of populations. In other words, this new knowledge should act as a true “friend,” not a “foe”.

## References

[B1] Dai M. Wang L. Luo J. (2025). Application of shear wave elastography in the assessment of renal cortical elasticity in patients with hypertension. Front. Med. (Lausanne) 12, 1624558. 10.3389/fmed.2025.1624558 41080934 PMC12510936

[B2] Du R. Yuan J. Huang Y. Jiang G. Duan Z. Yang H. (2025). Sarcopenia is not associated with hypertension, but sarcopenic obesity increases risk of hypertension: a 7-year cohort study. Front. Public Health 12, 1479169. 10.3389/fpubh.2024.1479169 39882123 PMC11774739

[B3] Fang Z. Huang T. Zhang Q. Shi L. Huang R. (2025). The impact of depression on mortality among older adult patients with hypertension: a systematic review and meta-analysis. Front. Public Health 13, 1603785. 10.3389/fpubh.2025.1603785 40823226 PMC12350467

[B4] Jarocki M. Green S. Wu H. H. L. Chinnadurai R. (2025). Cardiorenal syndrome in the elderly: challenges and considerations. Geriatr. (Basel) 10, 104. 10.3390/geriatrics10040104 40863571 PMC12385558

[B5] Kebede H. B. Yosef T. Bilchut A. H. Workie S. G. Shifera N. Mezgebu A. D. (2024). Self-care practices and associated factors among hypertensive patients at public hospitals in north shewa zone, Ethiopia. Front. Med. (Lausanne) 11, 1482061. 10.3389/fmed.2024.1482061 39540043 PMC11557392

[B6] Ronco C. (2011). Cardio-renal syndromes: from foggy bottoms to sunny hills. Heart fail. Rev. 16, 509–517. 10.1007/s10741-011-9226-6 21259069

[B7] Shangguan Q. Yang J. Li B. Chen H. Yang L. (2024). Association of the hemoglobin glycation index with cardiovascular and all-cause mortality in individuals with hypertension: findings from NHANES 1999-2018. Front. Endocrinol. (Lausanne) 15, 1401317. 10.3389/fendo.2024.1401317 38915892 PMC11194314

[B8] Wang Y. Chen Z. Fan H. Cao S. Wang X. Niu T. (2025). Key influencing factors analysis on life satisfaction among Chinese older adults with hypertension: a national cross-sectional survey. Front. Public Health 13, 1569935. 10.3389/fpubh.2025.1569935 40356825 PMC12066345

[B9] Zeng X. Xiao C. Xu W. Zeng Q. Wu J. Luo J. (2025). Estimated pulse wave velocity as a potential predictor of albuminuria in hypertension. Front. Med. (Lausanne) 12, 1636846. 10.3389/fmed.2025.1636846 40832107 PMC12358374

[B10] Ziegler K. A. Engelhardt S. Carnevale D. McAlpine C. S. Guzik T. J. Dimmeler S. (2025). Neural mechanisms in cardiovascular health and disease. Circ. Res. 136, 1233–1261. 10.1161/CIRCRESAHA.125.325580 40403111

